# Dietary Fiber Intake and Risk of Pancreatic Cancer: Systematic Review and Meta-Analysis of Observational Studies

**DOI:** 10.3390/ijerph182111556

**Published:** 2021-11-03

**Authors:** Daniele Nucci, Omar Enzo Santangelo, Sandro Provenzano, Cristina Fatigoni, Mariateresa Nardi, Pietro Ferrara, Vincenza Gianfredi

**Affiliations:** 1 Nutritional Support Unit, Veneto Institute of Oncology IOV-IRCCS, 35128 Padua, Italy; daniele.nucci@iov.veneto.it (D.N.); mariateresa.nardi@iov.veneto.it (M.N.); 2 Regional Health Care and Social Agency of Lodi, ASST Lodi, 26900 Lodi, Italy; omarenzosantangelo@hotmail.it; 3 Local Health Unit of Trapani, ASP Trapani, 91100 Trapani, Italy; provenzanosandro@hotmail.it; 4 Department of Pharmaceutical Sciences, University of Perugia, 06122 Perugia, Italy; cristina.fatigoni@unipg.it; 5 Center for Public Health Research, University of Milan—Bicocca, 20900 Monza, Italy; 6 Value-Based Healthcare Unit, IRCCS MultiMedica, 20099 Sesto San Giovanni, Italy; 7 School of Medicine, University Vita-Salute San Raffaele, 20132 Milan, Italy; gianfredi.vincenza@hsr.it; 8 CAPHRI Care and Public Health Research Institute, Maastricht University, 6211 Maastricht, The Netherlands

**Keywords:** cancer risk, diet, dietary fiber, meta-analysis, pancreas, pancreatic cancer, systematic review

## Abstract

The burden of pancreatic cancer varies greatly across countries, with the number of deaths, incident cases, and disability-adjusted life years more than doubling in recent years, and with high-income countries having the highest incidence and mortality rates. We conducted this systematic review with meta-analysis with the goal of summarizing the current evidence on dietary fiber intake and its role in reducing the risk of pancreatic cancer, given the importance of identifying risk factors. This systematic review followed the guidelines of the Cochrane Collaboration and the Meta-analysis of Observational Studies in Epidemiology and the Preferred Reporting Items for Systematic Reviews and Meta-Analyses 2020. The structured literature search was conducted on PubMed/Medline and Scopus, combining free text words and medical subject headings. Our review contained 18 records at the end of the process. Our results show that dietary fiber intake reduces the risk of pancreatic cancer. When the analysis was differentiated according to the type of fiber considered, sub-grouped by gender (reduction of around 60% among women), and when case-control studies were conducted, the strength of the association increased. Clinicians and policymakers should improve interventions to raise the population’s awareness regarding the consumption of high-fiber diets, both in practice and in terms of public health policy.

## 1. Introduction

Pancreatic cancer has one of the worst prognoses among cancers, with a high case fatality rate and an overall 5-year survival rate of around 5% [1,2]. Although the epidemiological burden of pancreatic cancer varies greatly across countries, the most recent Global Burden of Diseases, Injuries, and Risk Factors Study found that the number of deaths, incident cases, and disability-adjusted life years attributable to the disease has more than doubled globally from 1990 to 2017, with the highest incidence and mortality rates recorded in high-income countries [3].

Cigarette smoking, alcohol consumption, chronic pancreatitis, obesity, and diabetes mellitus have all been identified as risk factors for this illness [4]. In terms of diet, some associations between individual foods or nutrients (e.g., red or processed meat, foods high in saturated fatty acids or fructose, etc.) and the risk of pancreatic cancer have been examined in the literature, but results are still scarce [5,6]. A recent systematic review on the role of dietary patterns suggested that those characterized by high consumption of fruit and vegetables, whole grains, low-fat foods, and antioxidant nutrients (for instance, vitamin C and beta carotene) may reduce the risk of pancreatic cancer [5]. These findings were also consistent with previous research linking the Mediterranean diet to a lower risk of pancreatic and other cancers [7], owing to a combination of nutrients and food components which seem to play a protective role.

Fiber intake has been ascertained as an essential component of a healthy diet [8]. The beneficial role of fiber may be attributable to physical, anti-inflammatory, and prebiotic mechanisms, while the health benefits of dietary fiber encompass both metabolic and overall health [9]. In particular, reliable associations have been observed between a higher dietary fiber intake and a lower risk of developing neoplasms, including certain gastrointestinal tumors, namely colon and rectal cancers, and colorectal adenoma [10,11,12]. The protective role of fiber has been attributed to a number of potential physical and biological effects, although the mechanisms involved remain unclear. Evidence so far available has suggested that fiber may have a favorable role in reducing insulin resistance and insensitivity, aside from conferring effective protection due to anti-inflammatory pathways, with a resulting positive effect on pancreatic carcinogenesis [10,13].

Given the considerable burden of pancreatic cancer and the importance of identifying risk factors that could be modified by healthy nutritional recommendations, we conducted a systematic review and meta-analysis with the goal of summarizing the current evidence on dietary fiber intake and its role in reducing the risk of pancreatic cancer.

## 2. Materials and Methods

This systematic review follows the Cochrane Collaboration [14] and the Meta-analysis Of Observational Studies in Epidemiology (MOOSE) guidelines [15]. The Preferred Reporting Items for Systematic Reviews and Meta-Analyses 2020 guidelines (PRISMA) [16] were used to report the process and results. The structured literature search was conducted on PubMed/MEDLINE and Scopus on 11 July 2021, combining free text words and medical subject headings (MeSH). Keywords were combined using the Boolean operators AND and OR. No time filter was applied. The full search strategy is available in Supplementary Table S1. The review protocol was registered in advance on PROSPERO, the International Prospective Register of Systematic Reviews (ID number: CRD42021267601).

### 2.1. Inclusion/Exclusion Criteria

Articles had to meet the following criteria to be considered eligible: (i) written in English; (ii) population: adults ≥18 years (both female and male); (iii) interventions and exposures: highest dietary fiber intake; (iv) comparators/control: lowest dietary fiber intake or no intake; (v) type of study: epidemiologic studies (case-control, cross-sectional, or cohort studies).

Exclusion criteria were (i) articles not published in English; (ii) people under the age of 18; (iii) full text not available; (iv) interventions and exposures: fiber supplementation; (v) comparators/control: fiber supplementation; (vi) type of study: review article, meta-analysis, trial, expert opinion, commentary, or article with no quantitative information or details. A detailed description of inclusion/exclusion criteria is reported in Supplementary Table S2.

### 2.2. Selection Process and Data Extraction

Two review authors (OES and VG) independently screened titles and/or abstracts of studies collected using the search strategy and those gathered from additional sources to identify research that would meet the inclusion criteria outlined above. Two members of the review team (OES and VG) independently assessed the downloaded full text of these studies for their eligibility. Any disagreements about the eligibility of particular studies were resolved through discussion with a senior reviewer (MN). In line with previous studies [17,18], a standardized form was used to extract data from the included studies for the assessment of research quality and evidence synthesis. As in previous reviews [19,20], the extraction form was pre-piloted on five randomly selected eligible studies. The following data were extracted: first author, year of publication, the country where the study took place, study period, study design, number of participants, age and gender, main population characteristics, amount of dietary fiber intake, fiber intake assessment tool, pancreatic cancer diagnostic tool, maximally-adjusted effect size measures along with the corresponding 95% confidence interval (CI), any possible funds received for conducting the original study, and conflicts of interest declared. Data extraction was independently performed by two authors (VG and DN), and discrepancies were resolved through discussion (with a third author where necessary).

### 2.3. Strategy for Data Synthesis

A “flow diagram” charting the number of references at each stage in the review process was produced in line with the PRISMA 2020 guidelines [16]. The quantitative and qualitative results of the literature were summarized in narrative and descriptive tables. A full report was produced, which contained a narrative overview with a detailed description of the review methodology and findings.

### 2.4. Critical Appraisal

Two researchers (CF and PF) independently performed the critical appraisal using the Newcastle-Ottawa Scale (NOS) [21], a risk of bias assessment tool for observational studies that assigns up to nine points for the least risk of bias in three domains: (i) study group selection; (ii) comparability; and (iii) ascertainment of exposure and outcomes, respectively, for case-control and cohort studies. Based on these criteria and on the standard cut-off used in previous literature [22,23], studies were identified as being of high quality if the NOS was equal to or greater than 7 points.

### 2.5. Statistical Analysis

The effect size (ES) was calculated based on the odds ratio (OR), risk ratio (RR), hazard ratio (HR), and mean and sample size provided for each study. The ES was reported as OR with a 95% confidence interval. Where articles did not directly report the OR, but instead gave the number of events (cases) among those exposed and not exposed and the mean value for dietary fiber intake for each group, the ORs and CIs were calculated from these data and included in the meta-analysis. Subjects having the highest dietary fiber intake were compared to those with the lowest (or no) dietary fiber consumption. A meta-regression analysis was conducted in the case of homogeneity in the reporting of dietary fiber intake (in terms of unit of measure). We used both fixed and random models in the current meta-analysis. This approach was chosen because, in most cases, a fixed model is used when studies are determined to be similar. However, the random effect model is strongly recommended when heterogeneity is classified as moderate to high. Chi^2^ and I^2^ tests were used to assess the heterogeneity of the included studies. Heterogeneity was defined as high when I^2^ values > 75%, moderate when I^2^ values were between 50% and 75%, low for values ranging between 25% and 50%, and no heterogeneity for values below 25%. The graphical evaluation of the Funnel plot and the Egger’s regression asymmetry test were used to estimate potential publication bias; statistical significance was set at *p* < 0.10 [24]. A trim and fill method, searching missing studies to the right of overall, was used to adjust for publication bias where this was detected [25]. The meta-analysis was performed using the Prometa3^®^ software (Internovi, Cesena, Italy).

### 2.6. Subgroup and Sensitivity Analysis

In the case of studies using the same cohort (or study population), a sensitivity analysis was performed considering only the study with the highest quality score (QS) or with a larger sample size where the QS was equal, to rule out the possibility of overlapping effects. Moreover, the meta-analysis excluded studies with a computed OR. Furthermore, the sensitivity analyses only included studies with a follow-up (FU) equal to or greater than 9 years, validated tools to assess dietary fiber intake, type of diagnosis, and a QS greater than 7 [10]. A subgroup analysis based on study design (case-control v. cohort studies), type of fiber, and sex was also performed to corroborate the results obtained.

### 2.7. Cumulative Analysis

A cumulative analysis is a sequential meta-analysis, intended to evaluate how adding one study at a time modifies the ES. We ran three cumulative analyses: the first adding studies chronologically (from the first published analysis to the most recent publication); the second based on increasing sample size (from the smallest to the largest); and the third based on ascending dose of dietary fiber intake (from the lowest to the highest). These types of analyses improve the potential consistency of the results [26].

## 3. Results

### 3.1. Literature Search

**Figure 1:**
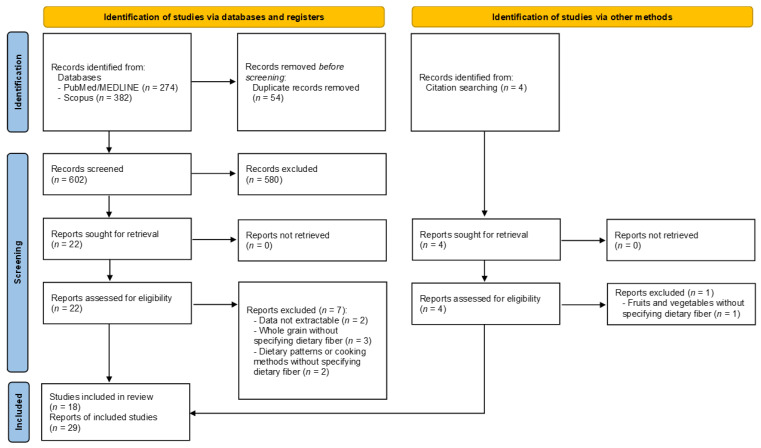
Flowchart of the study selection process.

The electronic searches on PubMed/MEDLINE and Scopus identified 274 and 382 records, respectively, for a total of 656 records. After screening the reference lists, four records were added, while 54 records were removed due to duplication. A total of 606 records were then assessed for eligibility. After screening the title and abstract, 580 records were eliminated because the topic was unrelated (*n* = 261), the articles were not original (*n* = 193), they were not written in English (*n* = 25), or were not based on human studies (in vitro (*n* = 65) and in vivo studies (*n* = 36)). The full text of the 26 records was downloaded, and eight records were excluded with reasons following an in-depth assessment. Supplementary Table S3 lists the detailed reasons for exclusion [27,28,29,30,31,32,33,34]. In brief, data could not be extracted, or results did not directly pertain to dietary fiber intake. At the end of the process, 18 records were included in our review [35,36,37,38,39,40,41,42,43,44,45,46,47,48,49,50,51,52]. However, because three records reported separate data for total soluble and insoluble fiber intake, and three records stratified data by gender, these were considered to be independent studies. Lastly, one record did not report the association between dietary fiber intake and pancreatic cancer as a risk, but as a number of events and non-events among those with a higher and lower intake. As a result, ORs were derived from these data. This estimated value was included in the overall pooled analysis, but was excluded from the sensitivity analysis. Figure 1 depicts the selection flowchart. There was a 12.3% disagreement between authors during the first screening. Table 1 and Table 2 illustrate the characteristics of the included studies, reported in alphabetical order. Table 1 reports the qualitative characteristics, while Table 2 focuses on the quantitative data.

### 3.2. Characteristics of Included Studies

Table 1 and Table 2 illustrate the characteristics of included studies, reported in alphabetical order. Table 1 reports the qualitative characteristics, while Table 2 focuses on the quantitative data. The first study was conducted in 1990 [42], while the most recent was published in 2019 [46]. The studies appear to be evenly spaced in time, with half of them published before the 2000s [35,37,39,41,42,44,45,48,51], and the other half published immediately after [36,38,40,43,46,47,49,50,52]. The majority of studies were conducted in Europe (*n* = 7; two studies published in Finland [49,50], one study conducted in Greece [45], in Italy [36], in Poland [51], in the Netherlands [37], and the United Kingdom [46]); five studies were conducted in the United States of America [38,40,43,48,52], and the remaining studies were conducted in Canada (*n* = 2) [39,42], Australia (*n* = 1) [35], China (*n* = 1) [44], and Japan (*n* = 1) [47]. Finally, one study was multicentric, with subjects enrolled from Australia, Canada, the Netherlands, and Poland [41].

Only three records were cohort studies [40,46,49], with 11 [40], 16 [49], and 17 [46] years of follow-up, respectively. The vast majority were case-control studies (*n* = 15) [35,36,37,38,39,41,42,43,44,45,47,48,50,51,52], of which one was a hospital-based case-control study [36], one was a combination of 5 different case-control studies [41], and one was a case-control from a subcohort study [50]. Almost all of the case-control studies (*n* = 10) matched cases and controls by sex and age, with three studies matching by residency as well [39,47,51], while the remaining two studies did not specify any type of matching [43,48]. Moreover, six studies enrolled patients who were alive or dead [37,41,42,43,48,51], while all the other case-control studies only involved live subjects.

The mean age of participants in virtually all of the included studies was 60 years old, with the exception of five studies in which the age of the participants was not reported [36,41,42,45,48]. Only men were enrolled in all but two of the studies [49,50]. Two-thirds of the total studies used a validated instrument to assess dietary fiber intake [36,38,39,40,43,46,47,49,50,51,52], while the remaining studies did not provide any information on validation. Half of the studies used a dietary interview conducted by trained personnel to assess dietary fiber intake [35,37,39,41,42,44,45,47,51], six studies used a Food Frequency Questionnaire (FFQ) [36,38,40,43,48,52], two studies used a Dietary History Questionnaire (DHQ), and one study used a food diary [46]. Almost all of the studies (*n* = 14) investigated dietary patterns from at least 1 year to 10 years before the pancreatic cancer diagnosis, regardless of the technique employed to assess dietary intake. Finally, due to the poor prognosis associated with the disease, the subjects’ proxies (caregivers, spouse, or the nearest relatives) were allowed to be interviewed in seven studies in order to estimate dietary fiber intake. In terms of diagnostic assessment, a histological/cytological or radiological confirmation was used in eight studies [36,39,42,43,44,45,51,52], followed by cancer registry (*n* = 7) studies [35,38,40,46,48,51], medical records (*n* = 2) [49,50], and clinical symptoms [47], while information was not reported in one study [41]. Funds were reported by 12 studies. However, conflicts of interest were not specified in 8 out of 12 studies, while conflicts of interest were not declared in the remaining studies [36,38,43,49].

### 3.3. Quality Assessment of Included Studies

The quality of the 18 studies ranged from 4 to 9, with a median value of 7. The assessment revealed a slightly higher quality level for cohort studies than for case-controls, and an overall increasing trend over time. The selection items, which consider the representativeness of the studies’ participants, mostly achieved high-quality criteria across the reports. On the other hand, serious flaws were discovered in the assessment of outcome/exposure, as well as in possible controlled factors. Quality evaluation results are presented in Table 2, while a complete overview based on the NOS checklist is illustrated in Supplementary Table S4.

### 3.4. Results of the Meta-Analysis and Sensitivity Analyses

**Figure 2:**
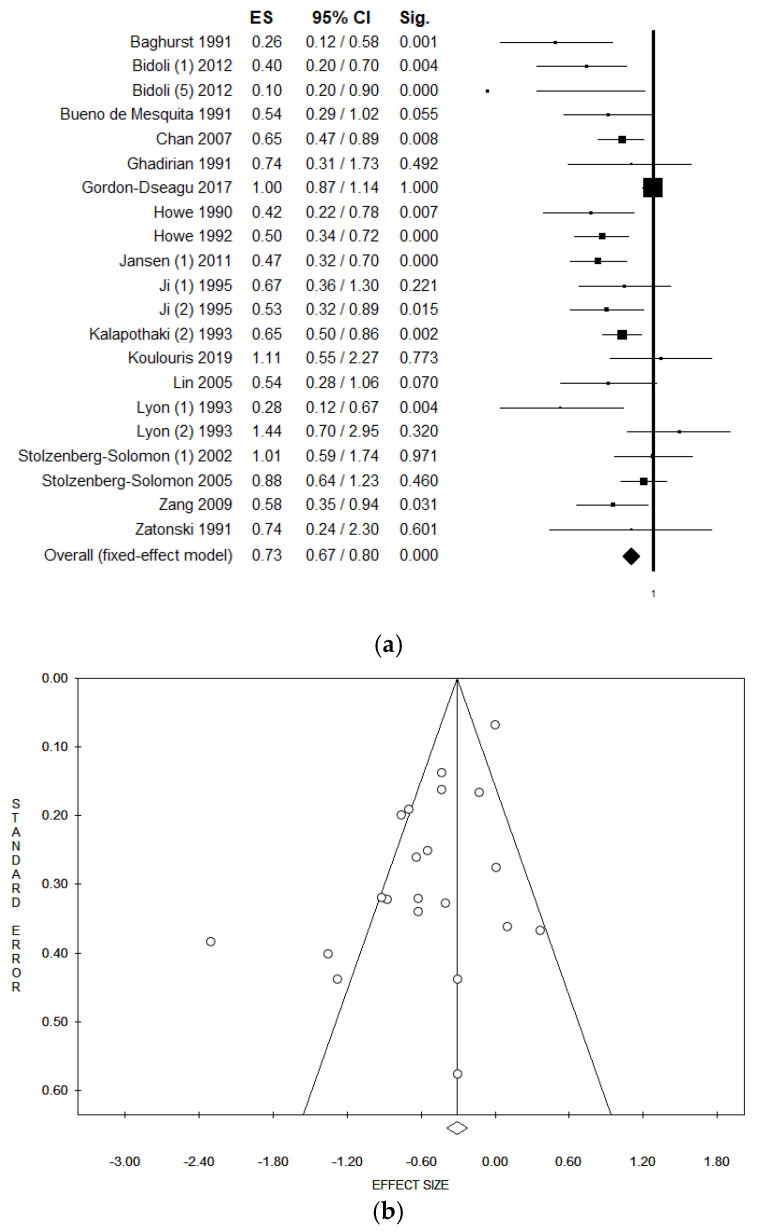
(**a**) Forest plot and (**b**) Funnel plot of the meta-analysis comparing dietary fiber intake (lower v. higher intake) and risk of pancreatic cancer (fixed effect model).

**Figure 3:**
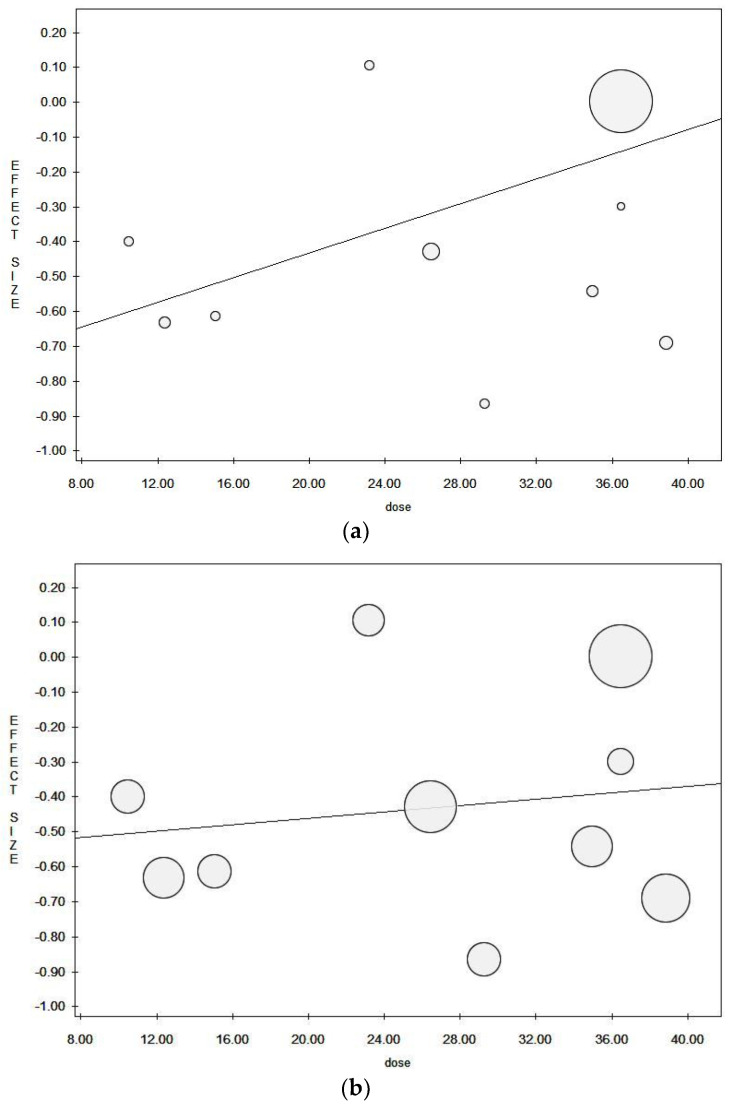
Meta-regression plot for (**a**) the fixed effect model and (**b**) the random effect model.

When pooling data in meta-analysis, higher dietary fiber intake was found to be associated with a significantly lower risk of pancreatic cancer in both fixed and random effect models (in the fixed effect model, pooled ES = 0.75 (95% CI = 0.69–0.82), *p*-value < 0.001; in the random effect model, pooled ES = 0.63 (95% CI = 0.53–0.76), *p*-value < 0.001; based on 343,120 subjects with a high statistical heterogeneity (Chi^2^ = 59.67, df = 19, I^2^ = 68.16%, *p*-value < 0.001)) (Figure 2a). As seen by the symmetry of the Funnel plot and corroborated by Egger’s linear regression test (Intercept −1.89, t = −3.12, *p* = 0.006), no publication bias was found in either the fixed or random effect models (Figure 2b). We eliminated the studies that came from the same population (or cohort) from the pooled analyses in order to exclude any potential overlapping effect. In particular, we removed the study conducted by Howe et al. in 1990 [42] because it could be considered a subgroup population of the data reported in Howe et al. 1992 [41]. However, the data remained unchanged (Table 3). We also eliminated the study by Stolzenberg-Solomon et al. 2002 [50], as the full cohort data were subsequently published. All studies with a calculated OR were also excluded. Additionally, in this case, there were no material changes in results (Table 3). As regards the dose meta-regression analysis, eight studies reported dietary fiber as grams per day (g/d) [38,39,40,41,42,44,46,47]. The meta-regression plots using fixed effect and random effect models are reported in Figure 3a,b, respectively. Results in the fixed effect model show a weak inverse borderline non-significant correlation between a higher intake of dietary fiber and the risk of pancreatic cancer (Y = −0.79, z = −0.02, *p* = 0.175). Results were not confirmed in the random effect model (Y = −0.55, z = −0.01, *p* = 0.662). There were no material changes in results when the analysis was restricted to those studies that used a validated dietary assessment tool, establishing a significant association between a higher intake of fiber and a lower risk of pancreatic cancer in the fixed and random effect models (Table 3). When considering the method used to diagnose pancreatic cancer, and only including those which used the cancer registry, results showed a borderline non-significant inverse association with a reduced risk of pancreatic cancer for higher dietary fiber intake (Table 3), with moderate heterogeneity. A statistically significant association between higher fiber intake and lower risk of pancreatic cancer was found in both the fixed and random effect models when only studies with QS > 7 were included. Heterogeneity in this analysis stood at approximately 65% (Table 3). In the sensitivity analysis involving studies that only interviewed pancreatic cancer patients (excluding those studies that allowed subjects’ proxies to be respondents), a statistically significant association was found in both the fixed and random effect models.

### 3.5. Subgroup Analysis

In both the fixed and random effect models, the subgroup analysis based on soluble and insoluble fiber individually found a significant association between a higher intake of dietary fiber and a lower risk of pancreatic cancer (Table 3). However, results should be interpreted with caution, since only three studies were included [36,43,49]. In order to estimate the risk of incident pancreatic cancer, all cohort studies were combined together in a subgroup analysis by study design [40,46,49]. In this case, results showed a non-significant association between fiber intake and the risk of incident pancreatic cancer. No heterogeneity was detected when only cohort studies were evaluated. However, a significant association between a higher intake of dietary fiber and a lower risk of pancreatic cancer was detected when only case-control studies with low heterogeneity were included (Table 3). We also performed a subgroup analysis by gender. Only three studies reported data for women [36,44,48], whereas five studies reported data for men [36,44,48,49,50]. In this case, results showed a statistically significant association between a higher intake of fiber and a lower risk of pancreatic cancer in both fixed and random effect models for women, but this significance for men only emerged when the fixed effect model was applied (Table 3).

## 4. Discussion

The findings of our systematic review and meta-analysis revealed that dietary fiber intake has a preventive effect against pancreatic cancer risk, which remained consistently significant across the sensitivity or subgroup analyses performed.

Worth noting is that the strength of the association increased when the analysis was differentiated based on the type of fiber considered (soluble or insoluble), and subgrouped by gender (highlighting a reduction of around 60% of pancreatic risk among women, compared to 30% lower risk among men), and study design (higher in case-control studies). However, results showed a weak inverse borderline association when the diagnosis relied on cancer registries, or when only cohort studies were considered. The strongest link between dietary fiber intake and pancreatic cancer was seen when only case-control studies were included rather than cohort studies, which is likely due to the small number of cohort studies found. Moreover, the period of the three cohort studies varied significantly (ranging from 10 to 30 years of follow-up). Cohort studies are also more prone to selection bias due to the lost-to-follow-up phenomenon, especially for extremely long studies. Even case-control studies, however, are prone to some limitations, such as recall bias, mainly because the difference between cohort and case-control studies is that in case-control studies, the exposure (in this case, dietary fiber intake) has already occurred in the past. Similarly, while the use of population-based cancer registries is of utmost importance for cancer surveillance, certain considerations (such as reporting delays and gaps) may limit their use. In particular, as suggested by Izquierdo et al., some disadvantages have been acknowledged in regard to the assessment of risk factors that are less detectable over longer time periods due to recall problems and difficulties in obtaining medical records from the distant past [53]. In terms of potential measurement bias, it should be taken into account that, generally speaking, dietary intake is usually influenced by some important methodological issue. Two of the main difficulties faced are measurement and, by extension, quantification of dietary intake. One of the most frequently criticized elements is the accuracy in quantifying dietary intakes, primarily due to the measurement methods’ inherent limitations (food diary, 24 h dietary recall, and food frequency questionnaire). These limitations are intrinsically linked to certain specific biases, including recall bias, misreporting, misclassification, and a variety of different forms of measurement error. The impact of these potential biases could be attributed to the slightly reduced beneficial effect of dietary fiber intake and risk of pancreatic cancer that we found when only studies with validated dietary assessment tools were included; or, when studies also including proxies (as husband/wife or caregivers) were excluded. Given that, in many cases, patients were no longer alive when the studies were performed, dietary assessments were conducted by interviewing patients or proxies in several studies. In this sensitivity analysis, we found that the reduction in pancreatic risk was marginally lower after accounting for a possible overestimation of dietary fiber intake reported by the proxies.

We also performed a sensitivity analysis stratified by type of fiber. Dietary fiber is defined in the literature as non-digestible carbohydrates plus lignin [54], which can be classified as “soluble” or “insoluble”, depending on its components (soluble fiber is mainly characterized by pectins, whereas, insoluble fiber is mainly based on cellulose). Despite the fact that this differentiation was mainly suggested to explore potential differences in biological mechanisms, data have shown that the two types of fibers function synergistically to improve health. This is supported by our findings, since our sensitivity analyses (which included studies assessing the effect of soluble fiber and the effect of insoluble fiber individually) found a higher reduction of pancreatic cancer risk. When both soluble and insoluble fibers were considered, the OR moved from 0.75 (in the pooled estimation) to 0.62 and 0.58, respectively. These results indicate that risk reduction increased from approximately minus 25% to minus 38–42%, with no particular difference between soluble and insoluble fiber. These findings could be potentially attributable to the higher quality assessment. Indeed, studies that differentiated between the two fibers were more recent studies, and data may have been more accurate as a result of the aim of differentiating between the fiber types. We noted that most of the included studies were quite old, with only four studies published during the last decade (from 2010 to 2020, *n* = 4/18). This aspect may have influenced the quality of available data due to fewer diagnostic tools as well as lower reporting accuracy, given that most scientific report guidelines are relatively new.

Finally, public health recommendations and healthy dietary guidelines all recommend a higher consumption of dietary fiber, even if no specific recommendations are available with regard to dietary fiber intake and pancreatic cancer. In fact, the most recent updated version of the World Cancer Research Fund/American Institute for Cancer Research (WCRF/AICR) report, which is used as a reference for cancer prevention through diet and nutrition, found limited evidence on dietary fiber intake and pancreatic cancer risk [6].

Nevertheless, the results of our systematic review and meta-analysis revealed that dietary fiber consumption protects against both prevalent and incident pancreatic cancer, also in line with literature so far available [55,56]. There are several potential biological mechanisms that can explain the beneficial effects of dietary fiber intake. Firstly, dietary fiber is associated with the stool bulk effect, which reduces carcinogen exposure in the intestinal lumen as well as secondary bile acid production by increasing transit speed [6]. Secondly, fiber disrupts the microbiota metabolism by stimulating the production of short-chain fatty acids (SCFA) through fiber fermentation. The production of these SCFA, in turn, lowers colonic pH and inhibits the growth of pathogenic microorganisms [13]. SCFA can also modulate inflammation [57], which has an effect on the risk of pancreatic cancer [58]. Dietary fiber intake also seems to improve glycemic control and other key risk factors such as abdominal obesity, metabolic syndrome, and insulin sensitivity [59], all of which are associated with an increased risk of cancer [60,61,62]. Moreover, low insulin sensitivity, insulin resistance, and type 2 diabetes are risk factors for pancreatic cancer [63]. Considering the above, it should be also mentioned that dietary fiber intake potentially correlates with other lifestyle and behavioral factors which might influence the risk for pancreatic cancer—namely obesity, alcohol intake, smoking—and a possible total synergic effect has to be also considered in further research [64].

Our findings have important implications on clinical practice, since recommended dietary fiber intake as part of healthy dietary pattern is also beneficial in pancreatic cancer prevention. This is especially true when considering the general public’s generally limited adherence to healthy eating recommendations [65]. Clinicians and policymakers should be aware of these findings in order to adopt interventions aimed at increasing population (and patient) awareness and the consumption of foods rich in fiber, both in practice and from a public health policy perspective [66,67].

### Strengths and Limitations

This study’s strengths and limitations should be acknowledged before generalizing the results of our systematic review with meta-analysis. To begin with, this is the most updated review assessing the association between dietary fiber intake and the risk of pancreatic cancer. In fact, Wang et al. published the first meta-analysis on the same topic in 2015, although only one cohort study and a lower number of case-control studies were obtained at that time [68]. Moreover, probably due to the lower number of included studies, they were unable to find a statistically significant association between dietary fiber and the risk of pancreatic cancer. They also performed a lower number of sensitivity analyses with no meta-regression analysis. We consider the important strengths of our work to be the several subgroups and sensitivity analyses performed, as well as the meta-regression analysis and the use of both fixed and random effect models. Another strength is that we followed the most updated and internationally-approved guidelines for conducting and reporting systematic reviews and meta-analyses. Several sensitivity and subgroup analyses were additionally conducted to improve the robustness and consistency of results, and a low or moderate heterogeneity was found, without publication bias.

The limitations should also be given due consideration. First of all, our results are affected by the intrinsic limitation of the original studies retrieved. As mentioned above, several of the retrieved studies have a medium or low-quality evaluation. It is worth also noting that the number of duplicates retrieved (*n* = 54) was lower than expected, likely because of the type of journals in which papers were published that are not indexed on more than one medical archive, that can confirm a medium or low-quality score of the included reports. In some cases, certainty with regard to dietary intake assessment is low due to the method used, or because proxies were also considered as valid respondents. However, independently of the respondent type, all dietary intake estimations derived from self-reported consumption which, as discussed above, even if validated, may be affected by some bias (such as recall and social desirability resulting in under or over-estimation). Since most of the retrieved studies were relatively old, it was not possible to perform a sensitivity analysis by pancreatic cancer subtype. Furthermore, some other limitations could be attributed to the review itself, as we only included articles published in English, and this might have affected the total amount of eligible studies. However, English is the commonly-accepted language within the scientific community and high-quality findings are usually published in international journals that only accept papers in the English language.

## 5. Conclusions

To conclude, our results demonstrate the beneficial effect that dietary fiber intake has in reducing the risk of pancreatic cancer. This protective role is particularly evident when considering the prevalence of pancreatic cancer, rather than the incidence. However, this result may be due to the very low number of cohort studies retrieved. Future prospective cohort studies should be encouraged in light of this. The results of this meta-analysis reveal that dietary fiber can protect both women and men. Furthermore, the robustness of results increased when a subgroup analysis by type of fiber was performed. Finally, a dose meta-regression analysis was conducted, which confirmed a weak inverse borderline association between a higher intake of fiber and a lower risk of pancreatic cancer. This systematic review’s pooled analysis, as well as several sensitivity and subgroup analyses, indicated the consistent and robust beneficial effect of dietary fiber intake on lowering the risk of pancreatic cancer.

## Figures and Tables

**Table 1:** Qualitative characteristics of included studies, reported in alphabetical order.

Author, Year [Ref.]	Country	Study Period	Study Design	Population Characteristics	Tool	Diagnostic Assessment	Cancer Type	Funds	Conflicts of Interest
Baghurst, 1991 [35]	Australia	1984–1987	Case-controls	Patients from major hospitals in Adelaide and from the cancer registry; controls selected from the electoral roll, matched by sex and age.	Personal dietary interview with no info on validation. Subjects’ proxies were also allowed to be interviewed. The food interview refered to 10 years previously (179 food items)	Cancer registry	Not specified	n.a.	n.a.
Bidoli, 2012 [36]	Italy	1991–2008	Hospital-based case-controls	Patients from two major hospitals in northen Italy; controls were selected from hospital patients and matched by sex, age, and study center	Personal interview by means of a validated 78-item FFQ referring to 2 years previously	Histology or cytology in 54.9% of cases, the others by ultrasound or tomography	Adenocarcinoma of the exocrine pancreas	yes	no
Bueno de Mesquita, 1991 [37]	The Netherlands	1984–1987	Case-controls	Live and dead patients residing in 70 municipalities; controls were selected from the general population and matched by sex and age	Personal dietary interview with no info on validation. Subjects’ proxies were also allowed to be interviewed. The food interview refered to 1 year previously	Clinical diagnosis retrieved from several medical records, including the cancer registry	Adenocarcinoma of the exocrine pancreas	n.a.	n.a.
Chan, 2007 [38]	USA	1995–1999	Case-controls	Live patients residing in six Bay Area counties able to complete an in-person interview; controls were selected from the general population and matched by sex and age	Validated 131-item semi-quantitative FFQ referring to 1 year previously	Cancer registry	Adenocarcinoma of the exocrine pancreas	yes	no
Ghadirian, 1991 [39]	Canada	1984–1988	Case-controls	Live patients from 19 hospitals in Montreal; controls were selected from the general population and matched for sex, age, and residency	Personal interview by means of NCIC 200-item validated FFQ	Histological, clinical, or radiological diagnosis	Not specified	yes	n.a.
Gordon-Dseagu, 2017 [40]	USA	1995–2006	Cohort	NIH-AARP Diet and Health study	Validated self-administered 37-item FFQ referring to 10 years previously	Cancer registry	Adenocarcinoma of the exocrine pancreas	n.a.	no
Howe, 1990 [42]	Canada	1983–1986	Case-controls	Live and dead patients diagnosed in 20 Toronto hospitals; controls were selected from the general population and matched by sex and age	Personal dietary interview with no info on validation. Subjects’ proxies were also allowed to be interviewed. The food interview refered to 1–2 years previously (200 food items)	Histology in 69% of cases, the rest were clinically or radiology confirmed	Not specified	n.a.	n.a.
Howe, 1992 [41]	Australia, Canada, the Netherlands, Poland	n.a.	Combination of data from 5 different case-control strudies	Live and dead patients; controls were selected from the general population and matched by sex and age	Personal dietary interview with no info on validation. Subjects’ proxies were also allowed to be interviewed. Different questionnaires among the studies	n.a.	Not specified	n.a.	n.a.
Jansen, 2011 [43]	USA	2004–2009	Case-controls	Live and dead patients; unrelated controls were selected from primary medical care	Validated self-administered 144-item FFQ referring to 5 years previously	Histology in 88%, medical records in 10%, and death certificate in 1%	Adenocarcinoma	yes	no
Ji, 1995 [44]	China	1990–1993	Case-controls	Live patients residing in Shangai; controls were selected from the general population and matched by sex and age	Personal dietary interview with no info on validation. The food interview refered to 5 years previously (86 food items)	Histology in 37%, surgery in 20%, and radiology in 43%	Not specified	n.a.	n.a.
Kalapothaki, 1993 [45]	Greece	1991–1992	Case-controls	Live patients diagnosed in 8 major teaching hospitals, with two control series. First control group hospitalized at the same hospital for other reasons; the second control group made up of residents who visited hospitalized patients and matched by sex and age	Personal dietary interview with no info on validation. The food interview refered to 1–2 years previously (110 food items)	Histological confirmation of cases	Not specified	yes	n.a.
Koulouris, 2019 [46]	UK	1993–2010	Cohort	EPIC-Norfolk	6-days food diary	Health records and cancer registry data reviewed by gastroenterologist	Pancreatic ductal adenocarcinoma	yes	n.a.
Lin, 2005 [47]	Japan	2000–2002	Case-controls	Patients 40–79 years of age, who lived in Aichi or Gifu Prefectures; controls were selected from the general population matched by sex, age, and residency	Personal dietary interview, validated. The food interview refered to 1–5 years before (97 food items)	Clinical symptoms, laboratory findings, and imaging	Not specified	yes	n.a.
Lyon, 1993 [48]	USA	1984–1987	Case-controls	Patients alive or death; controls were selected from US Health Care Financing Administration aged below 65 years	Personal dietary interview, no info on validation (32-item FFQ). Subjects’ proxies were also allowed to be interviewed.	Cancer registry	Not specified	yes	n.a.
Stolzenberg-Solomon, 2002 [50]	Finland	1985–1998	Case-controls from a subcohort study	ATBC Study	Self-administered, validated, 200-item DHQ	Medical records	Malignant neoplasm of the exocrine pancreas	yes	n.a.
Stolzenberg-Solomon, 2005 [49]	Finland	1985–2001	Cohort	ATBC Study	Self-administered, validated, 200-item DHQ	Medical records	Malignant neoplasm of the exocrine pancreas	yes	no
Zatonski, 1991 [51]	Poland	1985–1988	Case-controls	Live or dead patients residing in southwest Poland; controls were selected from electoral rolls and matched by sex, age, and residency	Personal dietary interview, validated. The food interview refered to 1–2 years previously (80 food items)	Medical and pathology records supported by cancer registry data	Malignant neoplasm of the exocrine pancreas	yes	n.a.
Zhang, 2009 [52]	USA	1994–1998	Case-controls	Live patients form all hospitals in 7 county metropolitan areas of Minnesota. Controls were selected from the general population of the same age, and matched by sex and age	Personal interview by means of a validated 153-item Willet FFQ referring to 1 year previously	Pathological confirmation	Malignant neoplasm of the exocrine pancreas	yes	n.a.

n.a.: not available; no: declared, but conflicts of interest absent; yes: declared and present; ATBC Study: Alpha-Tocopherol, Beta-Carotene Cancer Prevention Study; DHQ: dietary history questionnaire; FFQ: food frequency questionnaire; NIH-AARP Diet and Health Study: National Institute of Health—formerly, the American Association of Retired Persons; NCIC: National Cancer Institute of Canada; UK: United Kingdom; USA: United States of America.

**Table 2:** Quantitative characteristics of included studies, reported in alphabetical order.

Author, Year [Ref.] (Number of Stratified Analyses)	Total Sample ^	Sex	Age (in Years) Mean ± SD	Dietary Fiber Intake Mean ± SD	No. Subjects at the Highest Fiber Intake	Highest Dietary Fiber Intake g/d	Effect Size (95% CI) *p*	Adjustment	QS/9
Baghurst, 1991 [35]	Ca: 104 Co: 253	Ca: F = 52 Co: F = 111	≥50	n.a.	n.a.	n.a.	RR: 0.26 (0.12–0.58) *p* < 0.001	TEn, alcohol, smoking	6
Bidoli, 2012 (1) [36]	Ca: 326 Co: 652	Ca: F = 152 Co: F = 304	Ca: 63 Co: 63	16.1 ± 5.7	n.a.	n.a.	OR 0.4 (0.2–0.7) *p* < 0.001	BMI, education, smoking, alcohol, DM, folate intake, TEn	6
Bidoli, 2012 (2) [36]				8.1 ± 2.7 Soluble fiber			OR 0.4 (0.2–0.7) *p* < 0.001		6
Bidoli, 2012 (3) [36]				7.9 ± 3.2 Insoluble fiber			OR 0.5 (0.3–0.8) *p* < 0.003		6
Bidoli, 2012 (4) [36]	Only F	Ca: F = 152 Co: F = 304	n.a.	n.a.			OR 0.3 (0.1–0.8) *p* = n.a.		6
Bidoli, 2012 (5) [36]	Only M	Ca: M = 174 Co: M = 348					OR 0.1 (0.2–0.9) *p* = n.a.		6
Bueno de Mesquita, 1991 [37]	Ca:164 Co: 480	Ca: F = 74 Co: F = 248	Ca: 66.9 Co: 64.8	n.a.	n.a.	n.a.	OR 0.54 (0.29–1.02) *p* = 0.75	Age, sex, response status, smoking, TEn	6
Chan, 2007 [38]	Ca: 532 Co: 1701	Ca: F = 241 Co: F = 818	21–85 (range for both groups)	n.a.	81	26.5	OR 0.65 (0.47–0.89) *p* = 0.02	Age, sex, TEn, BMI, race, education, smoking, DM	9
Ghadirian, 1991 [39]	Ca:179 Co: 239	Ca: F = 82 Co: F = 198	35–79 (range for both groups)	Ca: 24.0 ± 11.9 g/d Co: 26.4 ± 14.4 g/d	n.a.	36.6	RR: 0.74 (0.31–1.73) *p* = n.a.	Age, sex, smoking, response status, TEn	6
Gordon-Dseagu, 2017 [40]	301,772 Ca: 1322	Ca: F = 36.6% no Ca: F = 42%	Ca: 66.0 no Ca: 63.5	n.a.	Ca: 438	9.2–54.6	HR: 1.00 (0.87–1.14) *p* = 0.92	Sex, TEn, smoking, BMI, DM	8
Howe, 1990 [42]	Ca:249 Co: 505	n.a.	n.a.	n.a.	n.a.	>29.3	RR: 0.42 (0.22–0.78) *p* < 0.001	TEn, fiber intake, smoking	6
Howe, 1992 [41]	Ca: 808 Co: 1669	n.a.	n.a.	n.a.	n.a.	38.9	RR: 0.50 (0.34–0.72) *p* < 0.001	Nutrient variables, smoking	4
Jansen, 2011 (1) [43]	Ca: 384 Co: 983	Ca: F = 163 Co: F = 500	Ca: 67.0 Co: 65.8	n.a.	Ca:.56	n.a.	OR: 0.47 (0.32–0.70) *p* < 0.001 Total dietary fiber	Age, sex, TEn, BMI, smoking, alcohol	7
Jansen, 2011 (2) [43]					Ca:.60		OR: 0.58 (0.39–0.86) *p* < 0.001 Soluble fiber		7
Jansen, 2011 (3) [43]					Ca:.57		OR: 0.48 (0.33–0.71) *p* < 0.001 Insoluble fiber		7
Ji, 1995 (1) [44]	Ca: 325 Co: 1552	n.a.	F: Ca: 65 Co: 61	n.a.	n.a.	≥10.5	F: OR: 0.67 (0.36–1.30) *p* = 0.26	Age, income, smoking, green tea, response status, TEn	7
Ji, 1995 (2) [44]			M: Ca: 63 Co: 62			≥12.4	M: OR: 0.53 (0.32–0.89) *p* = 0.01		7
Kalapothaki, 1993 (1) [45]	Ca: 181 Co: 181	n.a.	n.a.	n.a.	Ca: 38 Hospital Co: 43	n.a.	OR: 0.80 (0.64–1.00) *p* < 0.005	Age, sex, hospital, past residence, education, smoking, DM, TEn, nutrient variables	6
Kalapothaki, 1993 (2) [45]	Ca: 181 Co: 181				Resident controls: 55		OR: 0.65 (0.50–0.86) *p* < 0.001		6
Koulouris, 2019 [46]	Ca: 88 n Ca: 3970	Ca: F = 48 n Ca: F = 2230	Ca: 64.2 ± 7.8 n Ca: 59.3 ± 9.4	Ca: 14.8 ± 5.1 g/d n Ca: 15.0 ± 5.4 g/d	Ca: 18	23.2	HR: 1.11 (0.55–2.27) *p* = n.a.	Age, sex, smoking, DM, TEn	9
Lin, 2005 [47]	Ca: 109 Co: 218	n.a.	Ca: 64.7 ± 8.3 Co: 65.1 ± 8.6	n.a.	n.a.	>15.1	OR: 0.54 (0.28–1.06) *p* = 0.07	Age, smoking, nutrient variables	8
Lyon, 1993 (1) [48]	Ca: 60 Co: 166	Only F	n.a.	n.a.	Ca: 10	n.a.	OR 0.28 (0.12–0.67) *p* = 0.002	Age, smoking, coffee, alcohol	7
Lyon, 1993 (2) [48]	Ca: 85 Co: 191	Only M			Ca: 30		OR 1.44 (0.70–2.95) *p* = 0.90		7
Stolzenberg-Solomon, 2002 (1) [50]	Ca: 163 n Ca: 26,948	Only M	Ca: 58 n Ca: 57	Ca: 24 n Ca: 24 total dietary fiber	n.a.	n.a.	HR: 1.01 (0.59–1.74) *p* = 0.70	Age, smoking, TEn	9
Stolzenberg-Solomon, 2002 (2) [50]				Ca: 5.3 n Ca: 5.4 Soluble fiber			HR: 1.02 (0.56–1.70) *p* = 0.90	Age, smoking, TEn, energy-adjusted folate intake	9
Stolzenberg-Solomon, 2002 (3) [50]				Ca: 10.4 n Ca: 10.7 Insoluble fiber			HR: 0.95 (0.57–1.60) *p* = 0.99	Age, smoking, TEn, energy-adjusted folate intake	9
Stolzenberg-Solomon, 2005 [49]	Ca: 169 n Ca: 400	Only M	Ca: 58 n Ca: 56	Ca: 24.5 n Ca: 25.3	n.a.	n.a.	OR *: 0.88 (0.64–1.23) *p* = 0.460	None	7
Zatonski, 1991 [51]	Ca: 110 Co: 195	Ca: F = 42 Co: F = 106	Ca: 62.2 Co: 63.2	n.a.	n.a.	n.a.	RR: 0.74 (0.24–2.30) *p* = 0.87	Smoking, TEn	7
Zhang, 2009 [52]	Ca: 186 Co: 554	Ca: F = 75 Co: F = 240	Ca: 65.8 ± 10.9 Co: 66.5 ± 12.0	Ca: 22.4 ± 11.3 Co: 24.0 ± 10.4	Ca: 37 Co: 138	35.0	OR 0.58 (0.35–0.94) *p* = 0.021	Age, sex, race, education, smoking, alcohol	7

N.a.: not available; F: Female; M: male; * estimated; ^ The total sample and number of cases (Ca) and non-cases (n Ca) are reported for cohort studies, while both the number of cases (Ca) and controls (Co) are reported for case-control studies. BMI: Body Mass Index; DM: diabetes mellitus; g/d: grams per day; TEn: Total Energy intake; QS: quality score.

**Table 3:** Results of the sensitivity and subgroup analyses.

Analysis	Model	*N*. Studies Included	ES	95% CI	*p*	Sample Size	I^2^	*p*	Intercept	Tau (t)	*p*
Excluding potential overlapping cohort	Fixed	19	0.74	0.67–0.80	<0.001		78.24	<0.001	−2.35	−3.29	0.004
	Random		0.58	0.46–0.72	<0.001						
Excluding studies with estimated OR	Fixed	19	0.72	0.66–0.79	<0.001		78.63	<0.001	−2.40	−3.47	0.003
	Random		0.55	0.43–0.69	<0.001						
Soluble fiber	Fixed	3	0.62	0.47–0.83	0.001	29,456	60.60	0.079	−0.11	−0.22	0.990
	Random		0.62	0.39–1.01	0.053						
Insoluble fiber	Fixed	3	0.58	0.45–0.75	<0.001	29,456	58.34	0.091	6.72	1.01	0.498
	Random		0.60	0.40–0.90	0.014						
Validated dietary assessment	Fixed	11	0.84	0.76–0.93	0.001	336,147	64.07	0.002	−1.60	−2.16	0.059
	Random		0.72	0.57–0.89	0.003						
Diagnosis by cancer registry	Fixed	7	0.90	0.80–1.01	0.086	305,496	76.04	<0.001	−1.51	−1.42	0.214
	Random		0.70	0.48–1.04	0.079						
Quality score ≥ 7	Fixed	13	0.84	0.76–0.92	<0.001		65.09	0.001	−1.50	−2.07	0.063
	Random		0.72	0.58–0.89	0.003						
Cohort studies (incidence)	Fixed	3	0.99	0.87–1.11	0.819	302,668	0.00	0.749	−0.13	−0.17	0.894
	Random		0.99	0.87–1.11	0.819						
Case-Control (prevalence)	Fixed	17	0.58	0.51–0.66	<0.001		30.57	0.113	−0.46	−0.56	0.583
	Random		0.57	0.49–0.67	<0.001						
Women	Fixed	3	0.45	0.28–0.71	0.001		38.04	0.199	−4.71	−2.04	0.290
	Random		0.42	0.23–0.77	0.005						
Men	Fixed	5	0.71	0.57–0.89	0.006		88.38	<0.001	−3.66	−0.83	0.468
	Random		0.60	0.30–1.21	0.154						
No Proxy respondent	Fixed	14	0.80	0.73–0.88	<0.001		61.59	0.001	−1.65	−2.48	0.029
	Random		0.69	0.58–0.83	<0.001						

## Data Availability

All data are available in the current paper and Supplementary Material.

## Supplementary Materials

The following are available online at https://www.mdpi.com/article/10.3390/ijerph182111556/s1, Table S1: Full search strategy, Table S2: Detailed description of inclusion/exclusion criteria, based on PECOS (Population, Exposure, Comparison, Outcomes, and Study design), Table S3: Reasons for exclusion after full-text assessment, Table S4: Quality assessment of the included studies, using the Newcastle-Ottawa Scale (NOS).
