# Supplementary material for: Dietary Fiber Intake and Risk of Pancreatic Cancer: Systematic Review and Meta-Analysis of Observational Studies
Source: Int J Environ Res Public Health. 2021 Nov 3;18(21):11556. doi: 10.3390/ijerph182111556 (PMC8583332; doi:10.3390/ijerph182111556)
Supplement: Supplementary file 1 [file ijerph-18-11556-s001.zip › ijerph-1425884-supplementary.pdf]

Table S1: Full search strategy

Set	Database (PubMed/Medline)
	("Dietary Fibers"[All Fields] OR "Fibers"[Title] OR "Fiber"[Title] OR "fibre"[Title] OR "fibres"[Title] OR "Wheat Bran"[Title/Abstract] OR "Wheat Brans"[Title/Abstract] OR "Roughage"[Title/Abstract] OR "Roughages"[Title/Abstract] OR "grain"[Title/Abstract] OR "grains"[All Fields] OR "Bread"[MeSH Terms] OR "Edible Grain"[MeSH Terms] OR "Fruit"[MeSH Terms] OR "Vegetables"[MeSH Terms] OR "cereal"[Title/Abstract] OR "cereals"[Title/Abstract]) AND ("Pancreatic Neoplasms"[MeSH Terms] OR (("Pancreas"[Title/Abstract] OR "pancreatic"[Title/Abstract]) AND ("Cancer"[Title/Abstract] OR "tumor"[Title/Abstract] OR "neoplasm"[Title/Abstract] OR "cancers"[Title/Abstract] OR "tumors"[Title/Abstract] OR "neoplasms"[Title/Abstract])))

Set	Database (SCOPUS)
	((TITLE-ABS-KEY (dietary)) AND (fiber OR fibre OR fibers OR fibres OR "Wheat Bran" OR "Wheat Brans" OR grain OR grains OR cereal OR cereals)) AND ((TITLE-ABS-KEY (pancreas OR pancreatic)) AND (TITLE-ABS-KEY (cancer OR neoplasm)))

Table S2. Detailed description of inclusion/exclusion criteria, based on PECOS (Population, Exposure, Comparison, Outcomes and Study design)

**Search strategy**	**Details**
Inclusion criteria	P: adult population (female and male)<br>I: questionnaire/interview measuring dietary fibre intake<br>C: the highest vs the lowest intake<br>O: risk of pancreatic cancer (if any)<br>S: primary observational studies (cohort, case-control, cross-sectional)
Exclusion criteria	P: infants, children and adolescents<br>I: no assessment of dietary intake<br>O: other outcomes not related to pancreatic cancer risk<br>S: not original papers (opinion paper, review article, commentary, letter, article without quantitative data, book chapter)
Language filter	English
Time filter	No filter (from inception)
Database	PubMed/Medline; Scopus

Table S3. Reasons for exclusion after full-text assessment.

Author year [Ref]	Number of studies	Reasons for exclusion
Silverman, 1998 [27]; Jarosz, 2012 [28]	2	Data not extractable
Chatenoud, 1998 [29]; La Vecchia, 2003 [30]; Schacht, 2021 [31]	3	Whole grain without specifying dietary fiber
Zheng, 1993 [32]	1	Fruits and vegetables without specifying dietary fiber
Fraser, 1999 [33]; Ghorbani, 2015 [34]	2	Dietary patterns or cooking methods without specifying dietary fiber

Table S4. Quality assessment of the included studies, using the Newcastel-Ottawa Scale (NOS).

**Author, year [Ref]**	**Selection**				**Comparability**		**Outcome/Exposure^**			**Total score**
	**Item 1**	**Item 2**	**Item 3**	**Item 4**	**Item 5a**	**Item 5b**	**Item 6**	**Item 7**	**Item 8**	
Baghurst, 1991 [35]	*	*	*	*	-	*	-	*	-	6
Bidoli, 2012 [36]	*	-	-	*	*	*	-	*	*	6
Bueno de Mesquita, 1991 [37]	-	*	*	*	*	*	-	*	-	6
Chan, 2007 [38]	*	*	*	*	*	*	*	*	*	9
Ghadirian, 1991 [39]	*	-	*	*	*	*	-	*	-	6
Gordon-Dseagu, 2017 [40]	*	*	*	*	-	*	*	*	*	8
Howe, 1990 [42]	*	*	*	*	-	*	-	*	-	6
Howe, 1992 [41]	-	-	*	*	-	*	-	*	-	4
Jansen, 2011 [43]	*	*	-	*	*	*	*	*	-	7
Ji, 1995 [44]	*	*	*	*	*	*	-	*	-	7
Kalapothaki, 1993 [45]	*	*	-	*	*	*	-	*	-	6
Koulouris, 2019 [46]	*	*	*	*	*	*	*	*	*	9
Lin, 2005 [47]	*	*	*	*	*	*	-	*	*	8
Lyon, 1993 [48]	*	*	*	*	*	*	-	*	-	7
Stolzenberg-Solomon, 2002 [50]	*	*	*	*	*	*	*	*	*	9
Stolzenberg-Solomon, 2005 [49]	*	*	*	*	-	-	*	*	*	7
Zatonski, 1991 [51]	*	*	*	*	-	*	*	*	-	7
Zhang, 2009 [52]	*	*	*	*	-	*	-	*	*	7

^ Based on study design: for case-control the NOS assesses the exposure, for cross-sectional and cohort studies the outcome.
* Each star represents a high-quality criterion accomplished by the study.
